# Accelerating corneal wound healing using exosome-mediated targeting of NF-κB c-Rel

**DOI:** 10.1186/s41232-023-00260-y

**Published:** 2023-01-26

**Authors:** Wenbo Zhao, Xiaozhen He, Ruiling Liu, Qingguo Ruan

**Affiliations:** 1 Shandong First Medical University (Shandong Academy of Medical Sciences), Jinan, 250000 China; 2 Eye Institute of Shandong First Medical University, State Key Laboratory Cultivation Base, Shandong Provincial Key Laboratory of Ophthalmology, Qingdao, 266071 China; 3 Eye Institute of Shandong First Medical University, Eye Hospital of Shandong First Medical University (Shandong Eye Hospital), Jinan, 250021 China

**Keywords:** Corneal wound healing, Inflammation, NF-κB, siRNA, Exosome

## Abstract

The integrity of the corneal epithelium is essential for the maintenance of the physiological function of the cornea. Studies have found that inflammation greatly delays corneal wound healing. NF-κB c-Rel is preferentially expressed by immune cells and promotes the expression of inflammatory cytokines. In the current study, we sought to investigate whether c-Rel could be used as a potential therapeutic target for treating a corneal injury. Our studies reveal that expressions of c-Rel and its inflammatory targets are significantly increased in the cornea of mice with corneal injury. In addition, we find that c-Rel-deficient mice exhibit accelerated corneal wound healing and reduced expression of inflammatory cytokines. Further studies show that topical treatment on the corneal surface using nano-polymers or exosomes loaded with c-Rel-specific siRNA (siRel) can effectively accelerate regular and diabetic corneal wound healing. More importantly, we find that exosomes, as carriers of siRel, showed better efficacy than nano-polymers in treating corneal injury. We further demonstrate that exosomes secreted by mesenchymal stem cells can efficiently transfer siRNA into macrophages and dendritic cells but not T cells. Taken together, these results indicate that blocking c-Rel may represent an attracting strategy for the treatment of both regular and diabetic corneal injury.

**Supplementary Information:**

The online version contains supplementary material available at 10.1186/s41232-023-00260-y.

## Introduction

The cornea is a transparent tissue with no blood vessels and rich nerve endings, and as an important ophthalmic defense system, the cornea provides an important physical barrier against the infiltration of external substances [1]. Timely repair of corneal epithelial injury is essential since serious corneal injuries can result in inflammation, infection, ulceration, and even blindness [2]. Healing of corneal wounds maintains the integrity of the corneal epithelial surface, corneal transparency, and normal vision. Corneal wounding and healing are complex processes that include cell death and migration, cell proliferation and differentiation, and extracellular matrix remodeling [3, 4]. Studies have found that inflammation plays important roles in the pathogenesis of ocular inflammation [5]. It has been reported that SERPINA3K's inhibitory effect on the inflammation of injured cornea can be at least partially ascribed to the downregulation of TNF-α [6, 7]. Corneal wound healing was even more delayed when under diabetic condition [8]. Nearly half of diabetic patients exhibit clinical features such as increased sensitivity to injury and infection and delayed healing of the corneal epithelium, making it critical to improve corneal injury repair in diabetic patients [9].

Strategies for the treatment of corneal injury mainly include the removal of pathogenic factors, local treatment as a supplement to systemic therapy, promotion of corneal epithelial growth, and prevention of infection. However, current therapies in treating corneal injury are still not satisfactory [10]. Using artificial tears is simple and rapid, but requires multiple treatments [11, 12]. Anti-inflammatory drugs can efficiently treat the corneal injury but sometimes exhibit serious side effects and cannot be used for a long time [13]. Surgical treatment has been shown to be effective in treating severe corneal damage, but may also cause damage to the human body [14].

The mammalian nuclear factor (NF)-κB family consists of five members: c-Rel, RelA (p65), RelB, NF-κB1 (p50), and NF-κB2 (p52) [15]. Unlike other members that are constitutively expressed in multiple cell types, c-Rel is primarily expressed in activated lymphocytes and monocytes and regulates the expression of multiple inflammatory factors [16]. Because c-Rel-deficient mice develop normally and can effectively fight against viral and bacterial infections [17], c-Rel may be an ideal target for treating inflammatory diseases. We have previously shown that intravenous injection of nano-polymers loaded with c-Rel-specific small interfering RNA (siRNA) can effectively treat autoimmune diseases such as encephalomyelitis [18], arthritis [19], and psoriasis [20]. In addition, it has been reported that small molecule compound targeting c-Rel can effectively treat uveitis [21]. In the current study, we moved c-Rel-based therapy further forward by examining the efficacy of topical treatment of regular and diabetic corneal injury with nano-polymer and exosome-mediated targeting of c-Rel.

## Materials and methods

### Animals

Six- to 8-week-old male C57BL/6 mice were purchased from Beijing Vital River Laboratory Animal Technology Co., Ltd (Beijing, China). c-Rel-deficient mice (*c-Rel*^*−/−*^) were on the C57BL/6 background and purchased from Shanghai Model Organisms Center, Inc. (Shanghai, China). All mice were kept under pathogen-free conditions in the Shandong Provincial Key Laboratory of Ophthalmology and the Laboratory Animal Center of the State Key Laboratory Breeding Base jointly established by the province and the ministry. The protocol for the animal study was approved by the Animal Ethics Committee of the Institute of Ophthalmology of Shandong First Medical University. Animal use complies with the provisions of the Association for Research in Vision and Ophthalmology (ARVO) on the Declaration on Animal Use in Vision and Ophthalmology Research.

### Nucleotide sequences of siRNA

siRNA was synthesized by Guangzhou RiboBio Co., Ltd (Guangzhou, China) with the following sequences: control siRNA (siNC, sense: 5′-UUCUCC GAACGUGUCACGUdTdT-3′, anti-sense: 5′-ACGUGACACGUUCGGA GAAdTdT-3′); c-Rel-specific siRNA (siRel, sense: 5′-CAAC CGGACAUACCCGUCUdTdT-3′, anti-sense: 5′-AGACGGGUAUGUCCGG UUGdTdT-3′). 2’-O-Methyl oligonucleotide modification was introduced to siRNA. Fluorescein-tagged siRNA (FAM-siRNA) was synthesized by modification of the 3′-end of the sense strand of the c-Rel siRNA with fluorescein.

### In vivo corneal wound healing model

Mice were anesthetized using 0.6% sodium pentobarbital. Under a surgical microscope, a 2.5-mm diameter injury was first made in the corneal epithelium using 2.5-mm ring drill (Akihito Medical Devices, China) and then the corneal epithelium was removed using corneal rust ring remover (Alger, USA). The corneal epithelial wound healing process was evaluated by sodium fluorescein staining and examined under slit lamp microscope at the indicated time points. Image-J software (v1.8.0.112, National Institutes of Health, USA) was used to measure and calculate the corneal epithelial defect area.

### Type 1 diabetes model

To establish type 1 diabetes in mice using streptozotocin (STZ) (S0130, Sigma-Aldrich, USA), 6–8-week-old male C57BL/6 mice were first fasted for 6–8 h and then each mouse received intraperitoneal injection of STZ (10 mg/mL) at 50 mg/kg body weight. STZ treatment was performed daily for five consecutive days. One month after last STZ treatment, the blood glucose level was measured every 2 weeks using blood glucose meter (Sinocare, China). Mice with two consecutive readings of blood glucose levels above 16.7 mmol/L were considered diabetic. Mice will be used for further experiments 5–6 months after diagnosis of diabetes.

### Therapeutic treatment of corneal injury

The strategies to prepare siRNA-loaded nano-polymers or exosomes were illustrated in Figure S1. Briefly, to evaluate the therapeutic effects of nano-polymers loaded with c-Rel-specific siRNA (siRel) in corneal wound healing, negative control siRNA (siNC) or siRel was mixed with Entranster-in vivo transfection reagent (#18668-11-1, Beijing Ingern Biotechnology Co., Ltd, China) according to the manufacturer’s instructions and topically applied to the corneal surface (1.25 μg/eye) immediately after corneal epithelial scraping and at 12 h, 24 h, and 36 h hereafter (strategy 1). To evaluate the therapeutic effects of exosomes loaded with siRel in corneal wound healing, siNC or siRel was first transfected into human mesenchymal stromal cells (hMSCs, T0529, Abm, USA) using lipofectamine 3000 reagent (L3000015, Thermo Fisher, USA). After 6 h, replace the culture medium with exosome-depleted FBS (EXO-FBS-50A-1, SBI, USA) and continue to culture cells for 48 h. Exosomes in the culture supernatants were isolated using VEX Exosome Isolation Reagent (R601, Vazyme Biotech Co., Ltd, China) and re-suspended in PBS buffer (PH 7.4) in the absence of preservatives. The size of the isolated exosomes is between 40 and 120 nm in diameter as determined by electron microscopy. Exosomes were topically applied to the corneal surface (1.25 μg/eye) immediately after corneal epithelial scraping and at 12 h, 24 h, and 36 h hereafter (strategy 2). Alternatively, hMSCs were cultured in a culture medium with exosome-depleted FBS for 48 h and exosomes in the culture supernatant were isolated using VEX Exosome Isolation Reagent. siNC or siRel was then transferred into exosomes using Exo-Fect Transfection Kit (EXFT20A-1, System Biosciences, USA) according to the manufacturer’s instructions. Exosomes (1.25 μg/eye) were then re-suspended in PBS buffer and topically applied to the corneal surface immediately after corneal epithelial scraping and at 12 h, 24 h, and 36 h hereafter (strategy 3).

### RNA isolation and RT- PCR

Mouse corneas were isolated under a surgical microscope, and total RNA was isolated using TRIzol reagent following the manufacturer’s instructions (Life Technologies, USA). RNA samples were reversely transcribed using the primescript reverse transcription kit (Takara, JPN). The expression of mouse NF-κB family members were determined by quantitative RT-PCR using specific primers and Applied Biosystems 7500 system. When determining the relative level of gene expression, GAPDH was used as the internal control. The primer sequences are as follows: c-Rel-F: AGCACAGACAACAACCGGACATAC, c-Rel-R:TTCAATGTCCAGCAGCTGCTGTTC; RelA-F: AGGCTTCTGGGCCTTATGTG, RelA-R: TGCTTCTCTCGCCAGGAATAC; RelB-F: CCGTACCTGGTCATCACAGAG, RelB-R: CAGTCTCGAAGCTCGATGGC; p50-F: GGAGGCATGTTCGGTAGTGG, p50-R: CCCTGCGTTGGATTTCGTG; p52-F: GGCCGGAAGACCTATCCTACT, p52-R: CTACAGACACAGCGCACACT; Gapdh-F: GGTCGGTGTGAACGGATTTGG Gapdh-R: CCGTGAGTGGAGTCATACTGGAA).

### Enzyme-linked immunosorbent assay (ELISA) assay

For the preparation of tissue extract, corneas were isolated under a surgical microscope (Zeiss, Germany) and homogenized in PBS buffer using Tissue Lyser II (QIAGEN, USA) per manufacturer’s instructions. The concentration of TNF-α (#88-7324-77), IL-1β (#88-7013-77), IFN-γ (#88-7314-77), IL-23 (#88-7230-88), IL-12 (BMS6004), and IL-17A (#88-7371-77) in the corneal tissue extract was determined by ELISA per manufacturer’s instructions (eBioscience, USA).

### Western blotting

Western blot was used to determine c-Rel protein expression. Briefly, total protein from the cornea was prepared using RIPA lysis buffer containing protease inhibitors (#1861280, Thermo Fisher Scientific, USA), and a bicinchoninic acid (BCA) protein assay kit (P0012, Beyotime, China) was used for protein quantification. Protein extracts were separated by 10% SDS-PAGE gel and transferred to a PVDF membrane (#IPVH00010, Merck Millipore, USA). After blocking with 5% skim milk, the membrane was incubated overnight with anti-c-Rel (#67489S, 1:400 dilution, Cell Signaling Technology, USA) or anti-β-actin (M1210-5, 1:2000 dilution, Huaan Biologics, China). Membranes were then incubated for 1 h at room temperature with goat anti-mouse-HRP antibody (1:5000 dilution in blocking buffer, #SA00001-1, Proteintech, USA) or goat anti-rabbit-HRP antibody (#SA00001-2, 1:5000 dilution in blocking buffer, Proteintech, USA). Signals were detected by chemiluminescence assay using ChemiDoc Touch (Bio-Rad, USA) and Image Lab Touch software (v1.2.0.12, Bio-Rad, USA).

### Fluorescent labeling of exosomes with PKH26 or FAM-siRel

Exosomes isolated from the culture supernatant of hMSCs were reacted with PKH26 dye using PKH26 Red Fluorescent Cell Linker MiNi Kit (MINI26-1KT, Sigma-Aldrich, USA) according to the manufacturer’s instructions. Alternatively, exosomes were transfected with FAM-conjugated siRel using Exo-Fect Transfection Kit.

### Statistical analysis

Data were analyzed using GraphPad Prism 9.2.0 and expressed as mean ± SD. Multiple *t*-test was used to compare the significance of the differences between two groups. Differences were considered statistically significant at *P<0.05, **P<0.01, and ***P<0.001.

## Results

### Expressions of c-Rel and its inflammatory targets are increased in the cornea of mice with corneal injury

**Fig. 1:**
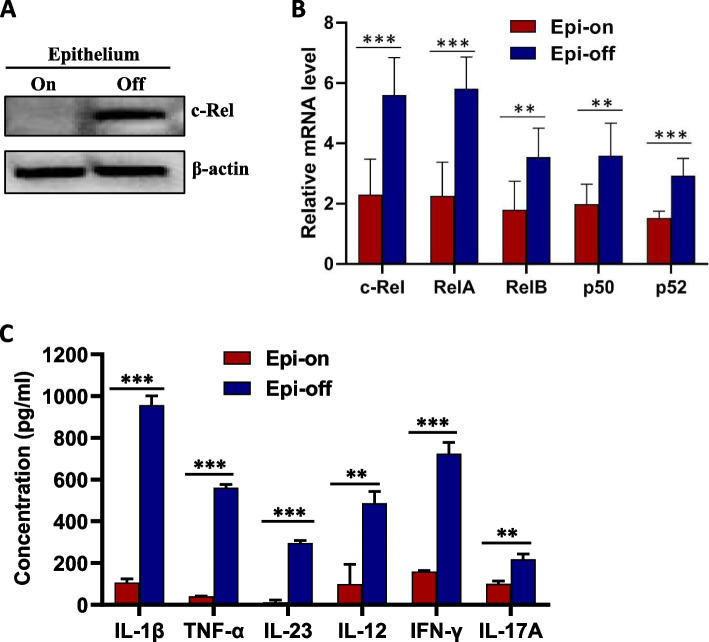
Expressions of c-Rel and its inflammatory targets are increased in the cornea of mice with corneal injury. Mouse corneal epithelium was either untreated (*n* = 6) or scraped (*n* = 6) as described in the “Materials and methods” section. Corneas were isolated 24 h after epithelial scraping. **A** Total proteins were prepared from the corneas and c-Rel expression was determined by Western blot. β-actin was used as internal control. The blot was cropped to improve the clarity of the image and full-length blot was provided in the supplementary materials. **B** Total RNA was isolated from the cornea and mRNA expression of all five NF-κB family members was determined by quantitative RT-PCR. **C** Corneal tissue extracts were prepared and the production of inflammatory cytokines as indicated in the figure was determined by ELISA. Epi on: epithelium not scraped. Epi off: epithelium scraped off. Results shown are representative of two independent experiments. **P<0.01, ***P<0.001

Inflammation is one of the important risk factors that contribute to delayed corneal wound healing. Since previous studies have shown that c-Rel is a major inflammatory mediator, we performed corneal wound healing in mice and examined the expression of c-Rel in the cornea before and after corneal injury. Our results showed that c-Rel protein expression (Fig. 1A) was increased in the cornea of mice with cornea injury (uncropped gel images were provided in Figure S2). We noticed that c-Rel expression was almost not detected in uninjured corneas. This is not surprising since c-Rel is mainly expressed by activated immune cells. We further showed that the mRNA expression of all five NF-κB family members were increased in the cornea of mice with cornea injury (Fig. 1B). To investigate whether increased c-Rel expression correlates with elevated expression of its targets, we examined the production of inflammatory cytokines regulated by c-Rel in the cornea extracts. Our results showed that the expression of inflammatory cytokines IL-1β, TNF-α, IL-23, IL-12, IFN-γ, and IL-17A was significantly increased in the cornea of mice with corneal injury (Fig. 1C). Taken together, these results indicate that c-Rel may delay corneal wound healing through promoting the expression of its inflammatory targets.

### Loss of c-Rel accelerates re-epithelialization of debrided mouse cornea

**Fig. 2:**
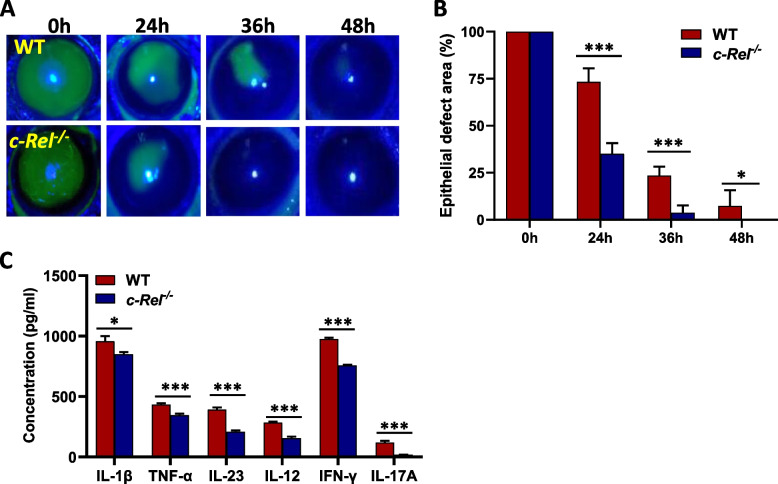
c-Rel-deficient mice exhibit accelerated corneal wound healing and reduced inflammatory cytokine production. The corneal epithelial wound healing model was established in both wild-type (*n* = 8) and c-Rel-deficient mice (*c-Rel*^*−/−*^) (*n* = 8) as described in the “Materials and methods” section. **A** Corneal epithelial wounds were stained with fluorescein sodium and examined under slit lamp microscope at the indicated time points. **B** Statistical analysis of corneal epithelial defect area using Image J software. **C** Corneal tissue extracts were prepared at 24 h after corneal epithelial scraping and the production of inflammatory cytokines as indicated in the figure was determined by ELISA. For **A** and **C**, results shown are representative of two independent experiments. For **B**, results shown are combined from two independent experiments. *P<0.05, ***P<0.001

To provide direct evidence that c-Rel is a potential risk factor for corneal wound healing, we established a corneal wound healing model in both wild-type and c-Rel-deficient mice. The central cornea of the wild-type and c-Rel-deficient mice were scraped to cause a 2.5-mm diameter injury, the repair of which was recorded at 0 h, 24 h, 36 h, and 48 h after scraping. There was a significant difference between wild-type and c-Rel-deficient mice in the rate of corneal epithelial healing (Fig. 2A). The corneal epithelial defect area in the c-Rel-deficient mice (24 h: 35.1%±5.6%; 36h: 3.7%±3.9%; 48 h: 0%±0%) was more significantly decreased than that in the wild-type mice (24 h: 73.3%±7.2%; 36h: 23.5%±4.7%; 48 h: 7.3%±8.4%) (Fig. 2B). The expression of c-Rel inflammatory targets in the cornea extracts from c-Rel-deficient mice was also significantly decreased (Fig. 2C). These results indicate that c-Rel indeed delays corneal wound healing and could be a potential therapeutic target for treating a corneal injury. Since our previous studies have shown that c-Rel-deficient mice are resistant to STZ- [22] and cyclophosphamide [23] -induced type 1 diabetes, we did not examine corneal wound healing in wild-type and c-Rel-deficient mice under diabetic condition.

### Topical treatment on the corneal surface with nano-polymers loaded with siRel accelerates corneal wound healing

**Fig. 3:**
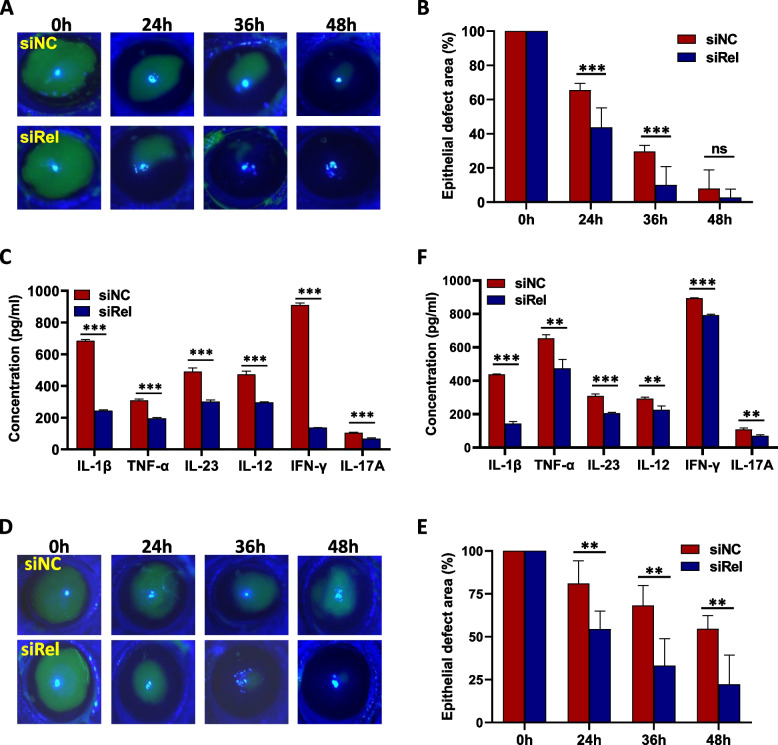
Topical treatment on the corneal surface with nano-polymers loaded with siRel can effectively accelerate corneal wound healing. **A**–**C** Corneal epithelial wound healing model was established in normal mice. Nano-polymers loaded with siNC (*n* = 8) or siRel (*n* = 8) were topically applied to the corneal surface immediately after corneal epithelial scraping and at 12 h, 24 h, and 36 h hereafter. Corneal epithelial wounds were stained with fluorescein sodium and examined under a slit lamp microscope at the indicated time points (**A**). Statistical analysis was performed on the corneal epithelial defect area using Image J software (**B**). Corneal tissue extracts were prepared at 24 h after corneal epithelial scraping and the production of inflammatory cytokines as indicated in the figure was determined by ELISA (**C**). **D–F** A corneal epithelial wound healing model was established in diabetic mice. Nano-polymers loaded with control siRNA (*n* = 6) or siRel (*n* = 6) were topically applied to the corneal surface as described in **A–C**. Corneal epithelial wound staining (**D**), statistical analysis of corneal epithelial defect area (**E**)**,** and detection of inflammatory cytokine production in corneal extracts (**F**) were then performed also as described in **A–C**. For **A**, **C**, **D,** and **F**, results shown are representative of two independent experiments. For **B** and **E**, results shown are combined from two independent experiments. *P<0.05, **P<0.01, ***P<0.001

We have previously shown that nano-polymers loaded with siRel can effectively downregulate c-Rel and its inflammatory targets in vitro [20]. In the current study, we evaluated the therapeutic efficacy of nano-polymers loaded with siRel in treating mouse corneal injury. As expected, c-Rel mRNA level was significantly reduced after treatment with siRel (Figure S3A). In addition, our results showed that there was a significant improvement in corneal wound healing after topical treatment on the corneal surface with siRel-loaded nano-polymers (Fig. 3A). The corneal epithelial defect area in the siRel-treated mice (24 h: 43.8%±11.3%; 36 h: 10.1%±10.8%) was more significantly decreased than that in the siNC-treated mice (24 h: 65.5%±4.0%; 36 h: 29.6%±3.6%) (Fig. 3B). The expression of c-Rel inflammatory targets in the cornea extracts from siRel-treated mice was also significantly decreased (Fig. 3C). Since high glucose toxicity has been implicated in the dysfunction of diabetic corneal wound healing, we next sought to investigate whether nano-polymers loaded with siRel can improve diabetic corneal wound healing. As expected, c-Rel mRNA level was significantly reduced after treatment with siRel (Figure S3B). In addition, our results showed that there was also a significant improvement in corneal wound healing after topical treatment on the corneal surface with siRel-loaded nano-polymers (Fig. 3D). The corneal epithelial defect area in the siRel-treated mice (24 h: 54.4%±10.6%; 36 h: 33.2%±15.8%; 48 h: 22.3%±17%) was more significantly decreased than that in the siNC-treated mice (24 h: 81.1%±13.3%; 36 h: 68.3%±11.7%; 48 h: 54.6%±7.7%) (Fig. 3E). Expression of c-Rel inflammatory targets in the cornea extracts from siRel-treated mice was also significantly decreased (Fig. 3F).

### Topical treatment on the corneal surface with exosomes loaded with siRel displays superior efficacy in accelerating corneal wound healing

**Fig. 4:**
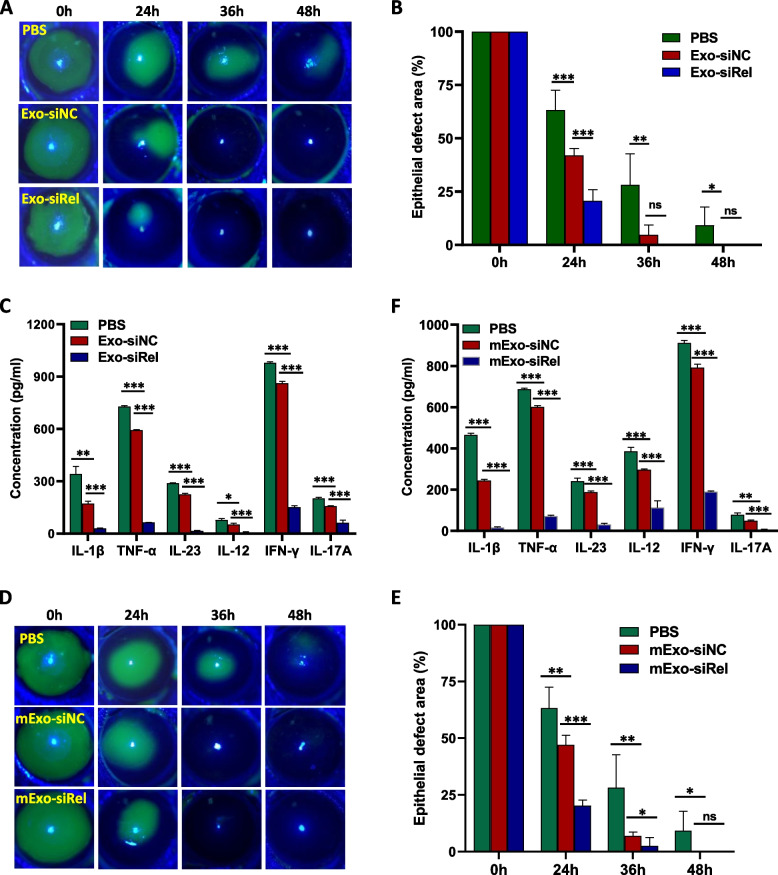
Topical treatment on corneal surface with siRel-loaded exosomes displays superior efficacy in accelerating conventional corneal wound healing. A corneal epithelial wound healing model was established in normal mice. **A**–**C** Exosomes were isolated from the supernatant of siNC (*n*=6) or siRel-transfected (*n*=6) hMSCs and topically applied to the corneal surface immediately after corneal epithelial scraping and at 12 h, 24 h, and 36 h hereafter. PBS-treated mice (*n*=6) were used as control. Corneal epithelial wounds were stained with fluorescein sodium and examined under a slit lamp microscope at the indicated time points (**A**). Statistical analysis was performed on the corneal epithelial defect area using Image J software (**B**). Corneal tissue extracts were prepared at 24 h after corneal epithelial scraping and the production of inflammatory cytokines as indicated in the figure was determined by ELISA (**C**). **D–F** hMSC-derived exosomes were transfected with either siNC (*n*=6) or siRel (*n*=6) and topically applied to the corneal surface as described in **A–C**. PBS-treated mice (*n*=6) were used as control. Corneal epithelial wound staining (**D**), statistical analysis of corneal epithelial defect area (**E**)**,** and detection of inflammatory cytokine production in corneal tracts (**F**) were then performed also as described in **A–C**. For **A**, **C**, **D**, and **F**, results shown are representative of two independent experiments. For **B** and **E**, results shown are combined from two independent experiments. *P<0.05, **P<0.01, ***P<0.001

Since recent studies have demonstrated the promise of using exosomes as drug delivery vehicles in vivo in animals and even in clinical situations, we next sought to investigate whether exosomes can be used to deliver siRel into the cornea and improve corneal wound healing. Exosomes containing siRel were generated by first transferring siRel into hMSCs and then exosomes (termed as Exo) were isolated from the culture supernatant. As expected, c-Rel mRNA level was significantly reduced after treatment with siRel (Figure S3A). In addition, our results showed that, compared with the PBS control group, there was a significant improvement in corneal wound healing after topical treatment on the corneal surface with siNC-loaded exosomes (Fig. 4A). The corneal epithelial defect area in mice treated with siNC-loaded exosome (24 h: 42.0%±3.3%; 36 h: 4.7%±4.5%; 48 h: 0%±0%) was more significantly decreased than that in mice treated with PBS (24 h: 63.2%±9.3%; 36h: 28.2%±14.5%; 48 h: 9.3%±8.5%) (Fig. 4B). Expression of c-Rel inflammatory targets in the cornea extracts from mice treated with siNC-loaded exosomes was also significant decreased (Fig. 4C). We further found that, compared with siNC-loaded exosomes, there was a significant improvement in corneal wound healing after topical treatment on the corneal surface with siRel-loaded exosomes (Fig. 4A). The corneal epithelial defect area in mice treated with siRel-loaded exosome (24 h: 20.7%±5.2%; 36 h: 0.1%±0.1%; 48 h: 0%±0%) was more significantly decreased than that in mice treated with siNC-loaded exosomes (24 h: 42.0%±3.3%; 36 h: 4.7%±4.5%; 48 h: 0%±0%) (Fig. 4B). Expression of c-Rel inflammatory targets in the cornea extracts from mice treated with siRel-loaded exosomes was also significantly decreased (Fig. 4C).

Alternatively, a different strategy was used to directly modify exosomes by isolating hMSC-derived exosomes first and then transfer siRel or siNC into them (termed as mExo). As expected, c-Rel mRNA level was significantly reduced after treatment with siRel (Figure S3A). In addition, our results showed that, compared with the PBS control group, there was a significant improvement in corneal wound healing after topical treatment on the corneal surface with siNC-loaded exosomes (Fig. 4D). The corneal epithelial defect area in mice treated with siNC-loaded exosome (24 h: 47.0%±4.2%; 36 h: 7.0%±1.6%; 48 h: 0%±0%) was more significantly decreased than that in mice treated with PBS (24 h: 63.2%±9.3%; 36h: 28.2%±14.5%; 48 h: 9.3%±8.5%) (Fig. 4E). Expression of c-Rel inflammatory targets in the cornea extracts from mice treated with siNC-loaded exosomes was also significantly decreased (Fig. 4F). We further found that, compared with siNC-loaded exosomes, there was a significant improvement in corneal wound healing after topical treatment on the corneal surface with siRel-loaded exosomes (Fig. 4D). The corneal epithelial defect area in mice treated with siRel-loaded exosomes (24 h: 20.2%±2.5%; 36 h: 2.6%±3.7%) was more significantly decreased than that in mice treated with siNC-loaded exosomes (24 h: 47.0%±4.2%; 36 h: 7.0%±1.6%) (Fig. 4E). Expression of c-Rel inflammatory targets in the cornea extracts from mice treated with siRel-loaded exosomes was also significantly decreased (Fig. 4F).

**Fig. 5:**
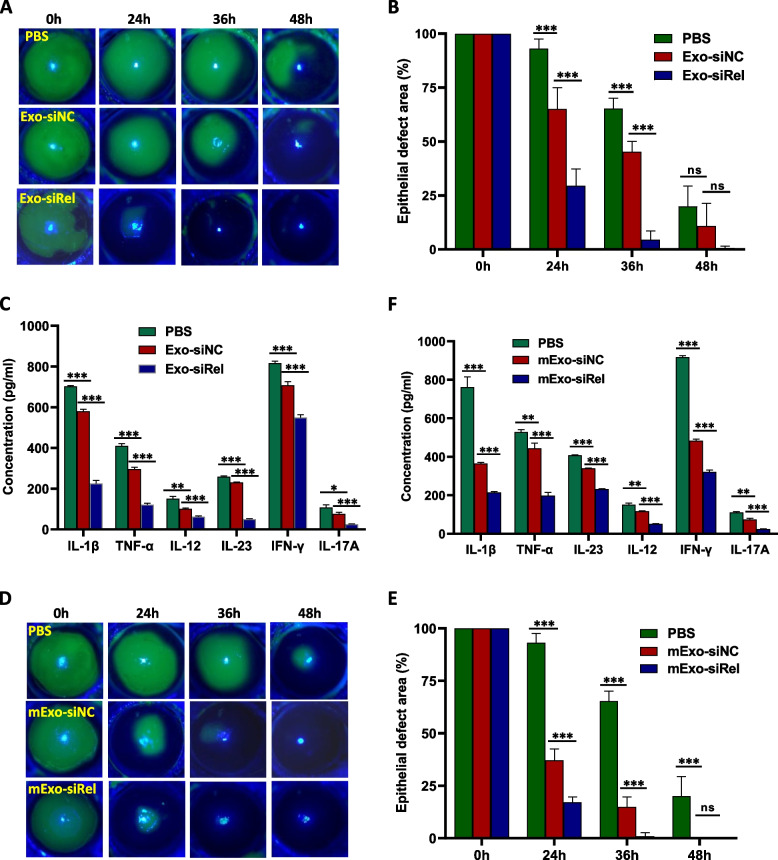
Topical treatment on the corneal surface with siRel-loaded exosomes displays superior efficacy in accelerating diabetic corneal wound healing. A corneal epithelial wound healing model was established in diabetic mice. **A–C** Exosomes were isolated from the supernatant of siNC (*n*=6) or siRel-transfected (*n*=6) hMSCs and topically applied to the corneal surface immediately after corneal epithelial scraping and at 12 h, 24 h, and 36 h hereafter. PBS-treated mice (*n*=6) were used as control. Corneal epithelial wounds were stained with fluorescein sodium and examined under a slit lamp microscope at the indicated time points (**A**). Statistical analysis was performed on the corneal epithelial defect area using Image J software (**B**). Corneal tissue extracts were prepared at 24 h after corneal epithelial scraping and the production of inflammatory cytokines as indicated in the figure was determined by ELISA (**C**). **D–F** hMSC-derived exosomes were transfected with either siNC (*n*=6) or siRel (*n*=6) and topically applied to the corneal surface as described in **A–C**. PBS-treated mice (*n*=6) were used as control. Corneal epithelial wound staining (**D**), statistical analysis of corneal epithelial defect area (**E**), and detection of inflammatory cytokine production in corneal extracts (**F**) were then performed also as described in **A–C**. For **A**, **C**, **D**, and **F**, results shown are representative of two independent experiments. For **B** and **E**, results shown are combined from two independent experiments. *P<0.05, **P<0.01, ***P<0.001

To further evaluate the therapeutic efficacy of exosome-mediated inhibition of c-Rel expression in accelerating diabetic corneal wound healing, we established a corneal wound healing model in diabetic mice and then treated them with PBS or exosomes containing either siNC or siRel. The results were similar to those obtained with normal mice. c-Rel mRNA was significantly reduced after treatment with siRel (Figure S3B). In addition, there was a significant improvement in corneal wound healing and reduction of inflammatory cytokine production after topical treatment on the corneal surface with exosomes isolated from the culture supernatant of hMSCs transfected with siRel (Fig. 5A–C) or hMSC-derived exosomes directly transfected with siRel (Fig. 5D–F). For exosomes isolated from the culture supernatant of hMSCs transferred with siRel, the corneal epithelial defect area in the Exo-siRel-treated mice (24 h: 29.4%±7.9%; 36 h: 4.5%±4.0%; 48 h: 0.7%±0.8%) was more significantly decreased than that in the Exo-siNC-treated mice (24 h: 65.1%±9.9%; 36 h: 45.2%±4.8%; 48 h: 10.9%±10.5%) and in mice treated with PBS (24 h: 93.1%±4.4%; 36 h: 65.3%±4.7%; 48 h: 20.0%±9.4%). For exosomes isolated from hMSCs and then transfected with siRel or siNC, the corneal epithelial defect area in the mExo-siRel treated mice (24 h: 17.1%±2.5%; 36 h: 1.0%±1.7%; 48 h: 0%±0%) was more significantly decreased than that in the mExo-siNC-treated mice (24 h: 37.1%±5.5%; 36 h: 14.9%±4.8%; 48 h: 0%±0%) and in mice treated with PBS (24 h: 93.1%±4.4%; 36 h: 65.3%±4.7%; 48 h: 20.0%±9.4%). It is worth noting that exosomes isolated from the culture supernatant of hMSCs transfected with siNC or hMSC-derived exosomes directly transfected with siNC also showed a significant improvement in corneal wound healing and reduction of c-Rel inflammatory targets expression compared with PBS control group.

**Fig. 6:**
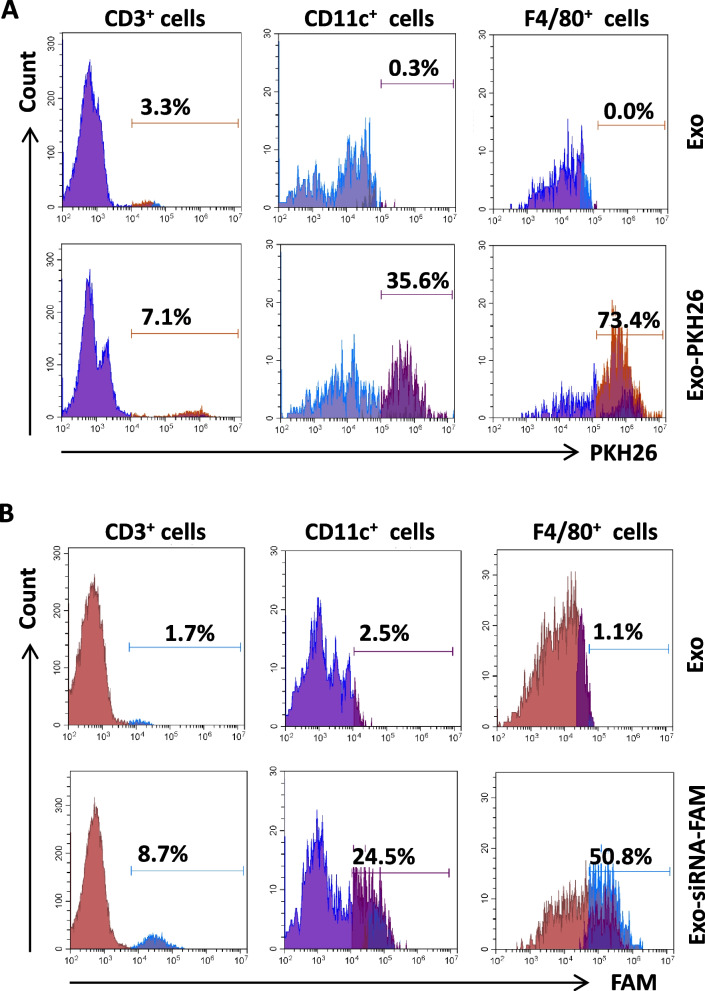
Exosomes secreted by mesenchymal stem cells can efficiently enter and transfer siRNA into macrophages and dendritic cells. Exosomes were isolated from the culture supernatant of hMCSs and labeled with PKH26 or transfected with FAM-conjugated siRel. Labeled exosomes were then co-cultured with mouse splenocytes for 24 h. The percentages of PKH26 (**A**) and FAM (**B**) positive T cells, dendritic cells, and macrophages were determined by staining cells with APC-conjugated antibodies against CD3, CD11c, and F4/80 and then analyzed by flow cytometry. Results shown are representative of two independent experiments

A previous study has shown that hMSC-derived exosomes can be efficiently taken up by human corneal epithelial cells and macrophages [24]. Since c-Rel is mainly expressed by activated immune cells, we then determined the efficiency of exosomes to enter or transfer siRNA into various types of immune cells. Exosomes isolated from the culture supernatant of hMSCs were labeled with either PKH26 or transfected with FAM-conjugated siRel and co-cultured with mouse splenocytes. Our results showed that hMSC-derived exosomes could not only efficiently enter macrophages (73.4%) and dendritic cells (35.6%) (Fig. 6A), but also can efficiently transfer siRel into macrophages (50.8%) and dendritic cells (24.5%) (Fig. 6B). However, the efficiency of exosomes to enter (7.1%) and transfer siRel (8.7%) into CD4^+^ T cells was relatively low (Fig. 6A, B).

## Discussion

Mammalian Rel/NF-κB family consists of five members: c-Rel, RelA, RelB, p50, and p52. NF-κB regulates the expression of a variety of genes related to inflammation, which makes it an important target for the treatment of inflammatory diseases [25–27]. However, because most Rel/NF-κB proteins are ubiquitously expressed and involved in a variety of important biological processes, drugs that block the entire NF-κB family may have significant side effects. Unlike other members of the NF-κB family, c-Rel is primarily expressed in activated immune cells and plays an important role in the inflammatory response. In addition, c-Rel-deficient mice develop normally and can effectively fight against viral and bacterial infections [17]. In the current study, we showed that expressions of c-Rel and its inflammatory targets are increased after corneal epithelial injury and c-Rel-deficient mice exhibit accelerated corneal injury repair. Furthermore, we demonstrated the therapeutic potential of c-Rel inhibition mediated by nano-polymers and exosomes in the treatment of both regular and diabetic corneal injuries.

siRNA can specifically silence gene expression from post-transcriptional level [28]. During recent years, siRNA-based RNA drugs have shown remarkable therapeutic effects in experimental studies and a variety of clinical diseases [29]. However, siRNA bare sequences are unstable and cannot efficiently penetrate cell membranes [30]. As a result, it is of great significance to design siRNA drug vectors for targeted delivery of siRNA in vivo. As a class of efficient delivery system, nanocarriers can extend the cycle time of siRNA in vivo, improve the stability and bioavailability of siRNA, and have already been approved for clinical use [31, 32]. However, nanocarriers are usually difficult to penetrate physiological barrier in vivo which results in low delivery efficiency [31]. In addition, nanocarriers are usually cytotoxic and easy to be cleared by plasma, which makes their therapeutic use face safety and efficacy challenges [33]. As a new generation of drug delivery vectors, exosomes are a subtype of extracellular vesicles (EVs) secreted virtually by all types of cells with a diameter range of 30–150 nm [34]. Exosomes exhibit high stability, high biocompatibility and permeability, low toxicity and immunogenicity, and the ability to encapsulate a wide variety of substances, making them attractive drug delivery vehicles [35].

There are several important findings from our current study. Firstly, hMSC-derived exosomes loaded with siRel displayed superior efficacy in accelerating corneal wound healing than exosomes loaded with siNC. Although damaged corneal epithelium can regenerate without any treatment in the corneal wound healing model we used and hMSC-derived exosomes can effectively accelerate corneal epithelial wound healing [24], our current study showed that combined with siRNA-mediated targeting of inflammatory factor c-Rel, hMSC-derived exosomes displayed superior efficacy in accelerating both conventional and diabetic corneal wound healing. Secondly, although nano-polymer-mediated c-Rel inhibition was effective in treating diabetic corneal injury, there was still a substantial delay in healing compared with normal mice treated with c-Rel-loaded nano-polymers. In contrast, exosome-mediated c-Rel inhibition accelerated diabetic corneal wound healing to almost the same extent as in normal mice. Since corneal epithelial healing is delayed in nearly half of diabetic patients, this finding is particularly important for the treatment of diabetic injury in clinic. Thirdly, we found that although exosomes isolated from siRNA-transfected hMSCs (Exo-siNC and Exo-siRel) and exosomes directly transfected with siRNA (mExo-siNC and mExo-siRel) were both effective in treating regular and diabetic corneal wound healing, exosomes directly transfected with siRNA showed better efficacy in treating diabetic corneal wound healing. While this phenomenon requires further investigation, it is possible that exosomes isolated from unmodified hMSCs (though exosomes were transfected with siRNA later) may modulate additional response under diabetic condition compared to exosomes isolated from siRNA-transfected hMSCs. It should be noted that for all the therapeutic effects observed, we are limited in our assessment of corneal damage and inflammation since we did not perform immunostaining.

Treating cornea-related diseases has several advantages over treating diseases of other organs. For example, the cornea is easily accessible thus allowing treatment by direct injection or topical application to the corneal surface. The relatively small size of the eye allows for the use of smaller drug doses compared to systemic administration. In addition, the ocular space is a partially isolated area, thus minimizing the unnecessary immune responses or toxicity to the whole body. Eye drops are the main form of topical administration due to good patient compliance and economic considerations. Drugs dissolved in eye drops are usually adsorbed by the corneal route and the conjunctiva route. However, due to the presence of a corneal barrier, the efficacy of the total administered drugs is less than 5% [36]. During recent years, increasing attention is being given to exosomes as a medium for drug delivery because of the limitations of traditional methods for the treatment of eye diseases. It has been reported that hMSC-derived exosomes can be taken up by corneal epithelium in vivo (after linear scratches). The distribution of exosomes throughout the mouse cornea can be detected even 4 h after topical treatment on corneal surface [24]. This result, in conjunction with our current findings, suggests that exosomes may be a promising vector for the treatment of corneal defects.

Although we have demonstrated that exosome-mediated targeting of c-Rel can successfully treat the corneal injury, there are still a few limitations about our current study. Firstly, we used Exosome Isolation Kit to isolate exosomes, which may not be suitable for clinical studies. In fact, although several methods can be used to isolate exosomes [37, 38], it remains a great challenge to prepare large amounts of exosomes with high quality for clinical use. Secondly, the exosomes we used lack targeting and therefore may reduce the efficiency of drug delivery in target cells, such as immune cells. In addition, inflammatory cytokines produced by T-cell are also involved in corneal injury [39, 40], yet our data showed that hMSC-derived exosomes cannot efficiently deliver drugs into purified mouse T cells in vitro. Since it has been shown that human MSC-derived exosomes can be efficiently taken up by human T cells in vitro [41], the reason why exosomes were poorly internalized by purified mouse T cells in vitro may be due to differences in the human versus the mouse system, as the exosomes used in this study were derived from immortalized human mesenchymal stromal cells. Notably, exosomes can modulate T cell response without being internalized by T cells, for example, through direct binding to immune suppressive protein ligands on T cells. In addition to their direct effects on T cells, exosomes can also modulate T cell response by affecting macrophage and dendritic cells. Thirdly, we performed exosome treatment every 12 h because exosomes have a relatively short half-life [42]. Combination with biocompatible materials such as hydrogels [43, 44] could allow the slow release of drugs and maintain the stability of exosomes, therefore reducing the frequency of exosome treatment.

## Conclusion

Our current study suggests that c-Rel could be a promising target for the treatment of the corneal injury. Although these results need to be confirmed in a clinical setting, they provide evidence that exosome-mediated targeting of c-Rel may represent an attracting strategy for the treatment of not only corneal injuries, but also other inflammatory corneal diseases.

## Supplementary Information

**Additional file 1: Figure S1.** Strategies used in this study to treat corneal injury. **Figure S2.** Uncropped images of c-Rel Western blot results. **Figure S3.** c-Rel mRNA level was significantly reduced after treatment with c-Rel-specific siRNA (siRel).

## Data Availability

All data generated or analyzed during this study are included in this manuscript.

## Abbreviations

siRNA: Small interfering RNA
NF-κB: Nuclear factor kappa-B
STZ: Streptozotocin
Exo: Exosome
siNC: Negative control siRNA
siRel: c-Rel-specific siRNA
BCA: Bicinchoninic acid
hMCSs: Human mesenchymal stromal cells
