# Supplementary material for: Accelerating corneal wound healing using exosome-mediated targeting of NF-κB c-Rel
Source: Inflamm Regen. 2023 Jan 26;43:6. doi: 10.1186/s41232-023-00260-y (PMC9881367; doi:10.1186/s41232-023-00260-y)
Supplement: Supplementary file 1 — Additional file 1: Figure S1. Strategies used in this study to treat corneal injury. Figure S2. Uncropped images of c-Rel Western blot results. Figure S3. c-Rel mRNA level was significantly reduced after treatment with c-Rel-specific siRNA (siRel). [file 41232_2023_260_MOESM1_ESM.pdf]

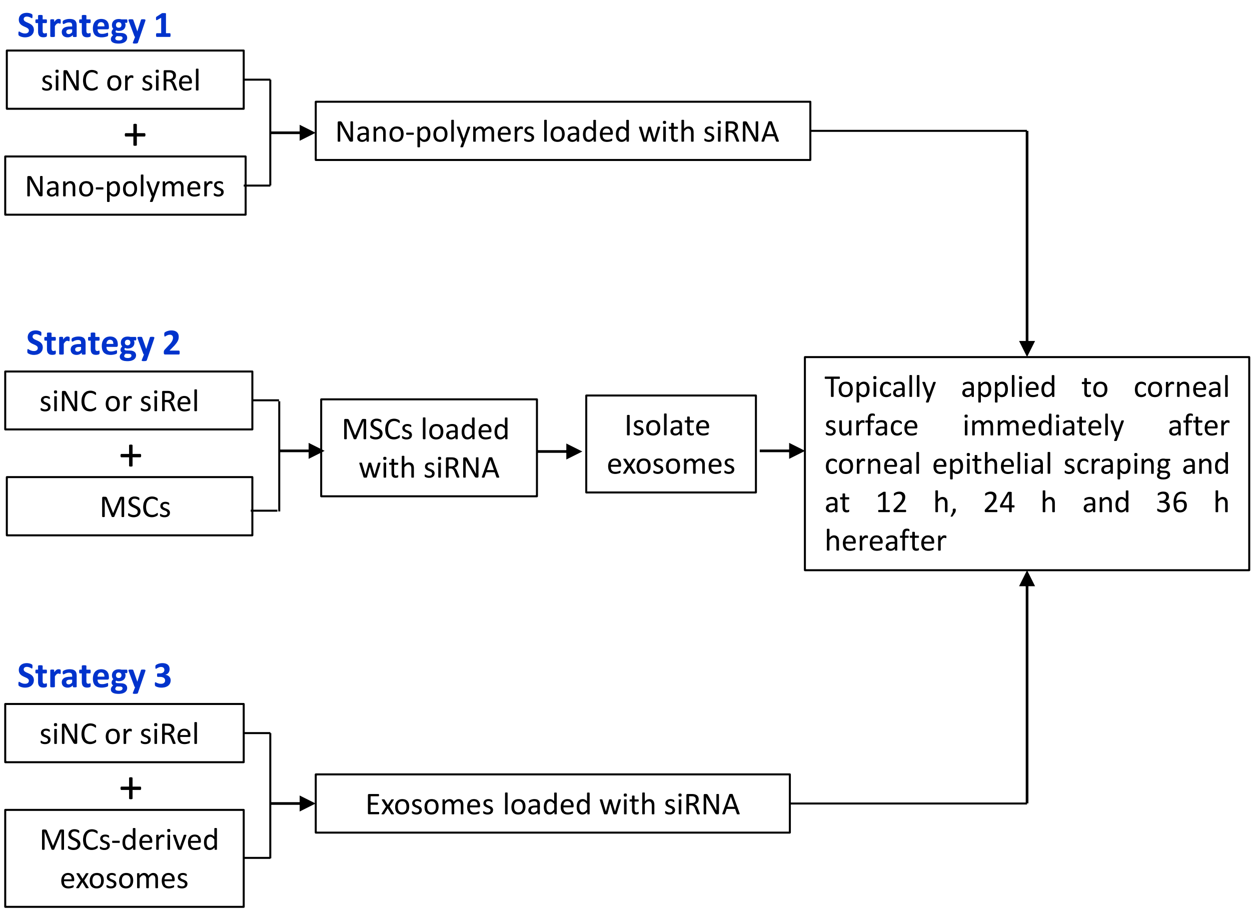

Figure S1. Strategies used in this study to treat corneal injury

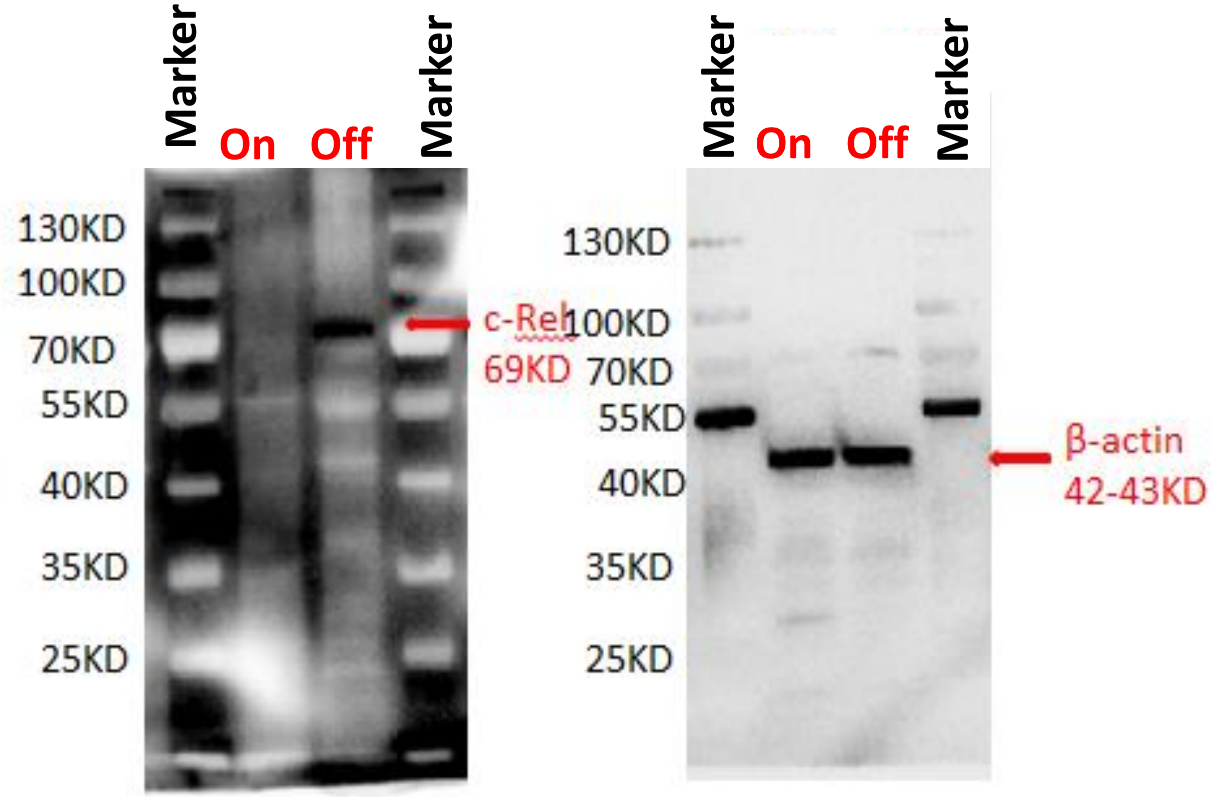

Figure S2. Uncropped images of c-Rel western blot results

A

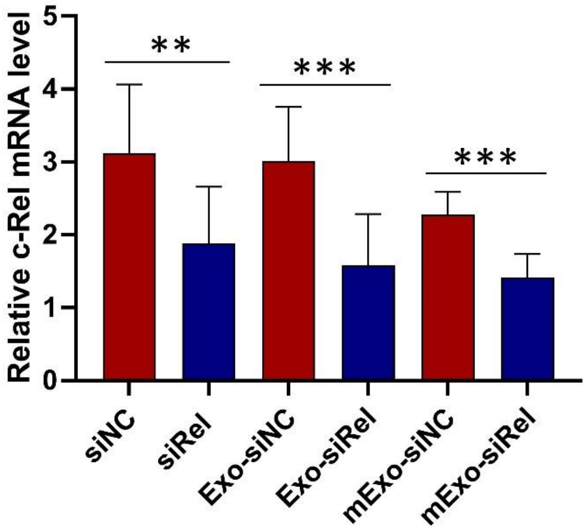

B

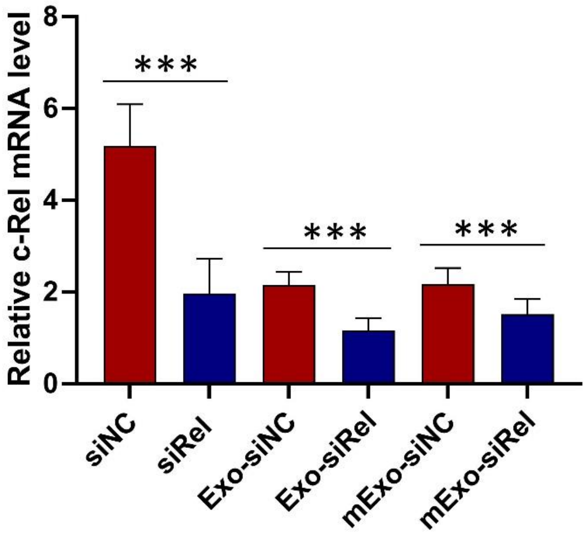

Figure S3. c-Rel mRNA level was significantly reduced after treatment with c-Rel-specific siRNA (siRel). Normal **(A)** (n=3) or diabetic mice **(B)** (n=3) were treated with nano-polymers loaded with siNC (siNC) or c-Rel-specific siRNA (siRel). Alternatively, mice were treated with exosomes loaded with siNC (Exo-siNC & mExo-siNC) or c-Rel-specific siRNA (Exo-siRel & mExo-siRel). After 24 h, c-Rel mRNA expression in the cornea was determined by quantitative RT-PCR. Results shown are representative of two independent experiments. **P<0.01, ***P<0.001.
